# Barriers to accessing health care in Nigeria: implications for child survival

**DOI:** 10.3402/gha.v7.23499

**Published:** 2014-03-14

**Authors:** Sunday A. Adedini, Clifford Odimegwu, Olusina Bamiwuye, Opeyemi Fadeyibi, Nicole De Wet

**Affiliations:** 1 Demography and Population Studies Programme, Schools of Public Health and Social Sciences, University of the Witwatersrand, Johannesburg, South Africa; 2 Demography and Social Statistics Department, Faculty of Social Sciences, Obafemi Awolowo University, Ile-Ife, Nigeria

**Keywords:** cultural barriers, physical barriers, access, health care, under-five mortality

## Abstract

**Background:**

Existing studies indicate that about one in every six children dies before age five in Nigeria. While evidence suggests that improved access to adequate health care holds great potential for improved child survival, previous studies indicate that there are substantial barriers to accessing health care in Nigeria. There has not been a systematic attempt to examine the effects of barriers to health care on under-five mortality in Nigeria. This study is designed to address this knowledge gap.

**Data and method:**

Data came from a nationally representative sample of 18,028 women (aged 15–49) who had a total of 28,647 live births within the 5 years preceding the 2008 Nigeria Demographic and Health Survey. The risk of death in children below age five was estimated using Cox proportional hazard models and results are presented as hazards ratios (HR) with 95% confidence intervals (CI).

**Results:**

Results indicate higher under-five mortality risks for children whose mothers had cultural barriers and children whose mothers had resource-related barriers to health care (HR: 1.44, CI: 1.32–1.57, *p*<0.001), and those whose mothers had physical barriers (HR: 1.13, CI: 1.04–1.24, *p*<0.001), relative to children whose mothers reported no barriers. Barriers to health care remained an important predictor of child survival even after adjusting for the effects of possible confounders.

**Conclusion:**

Findings of this study stressed the need for improved access to adequate health care in Nigeria through the elimination of barriers to access. This would enable the country to achieve a significant reduction in childhood mortality.

Globally, about 7 million under-five deaths were recorded in 2011 (1). About 41% of these deaths occurred in sub-Saharan Africa (2), even though the vast majority of the deaths are preventable using low-cost public health interventions (3). Unfortunately (4), preventable deaths of under-five children remain very high in sub-Saharan Africa due to poor access to timely and quality health care interventions (4). Despite the high potential that adequate health care interventions hold for the survival of young children, health care utilization has remained limited in many parts of sub-Saharan Africa (5). Although, utilization of modern health care is closely related to child survival, a number of barriers prevent many people from access. Consequently, poor utilization of health care services presents a daunting challenge to the attainment of the Millennium Development Goals (MDG) in many countries even as 2015 – the target year – draws closer. Kadobera et al. (6) observed that the coverage and quality of health care interventions for children require improvements for MDG 4 (reducing childhood mortality by 2015) to be achieved in many sub-Saharan African countries.

Generally, health care utilization is limited in sub-Saharan Africa (5) and studies have attributed this to many reasons. For instance, long distance to health care facilities has long been established as one of the barriers to health care utilization (6). Physical barriers to accessing health care facilities were found to be a determinant of child mortality in rural Tanzania (6). Frankenberg (7) noted that the proximity to a health care facility significantly decreases child mortality while a slight increase in the distance to a health care facility leads to a corresponding increase in child mortality risks. Moreover, studies have established that long distance to health care facilities causes delays in the decision to seek care (8, 9). This problem is particularly heightened in the rural areas of developing countries where the density of modern health care facilities is low and in settings where transportation systems and road infrastructures are poor (10).

Evidence shows that progress on childhood mortality reduction in most developing countries has witnessed a widening gap as well as a concentration of under-five deaths in the most deprived communities (11). One of the reasons given for this inequality in childhood mortality reduction is the growing inequality in access to health care across communities and regions, particularly in sub-Saharan Africa. In addition, there are uneven distributions of health care facilities and a dearth of health personnel across communities due to the scarcity of health funds (12) and due to health budget cutbacks (13) in many developing countries. Because health care facilities are widely dispersed, many patients have to travel long distances in order to receive treatment. Whitworth and Stephenson (14) noted that two neonates with similar characteristics may suffer different neonatal mortality risks if they are exposed to different antenatal and obstetric health care as a result of differences in access and quality of care. Harttgen and Misselhorn (15) argued that there would be differences in health outcomes of children from two communities that have contrasting characteristics. Community conditions have been noted to impact on health outcomes of individuals (16) through such levers as physical structures, social structures, and service provisions (17). For instance, children born or raised in communities that lack a health care facility are likely to suffer poorer health outcomes compared to those children from communities where good health facilities are available (18).

Moreover, other studies have established other barriers that could inhibit access, even if health care facilities and personnel are well distributed and available with high density. For instance, cultural practices such as the practice of purdah system (i.e. wife seclusion) have been cited (19) as a major barrier restricting women's access to health care in the Northern part of Nigeria (19, 20).

Whitworth and Stephenson (14) noted that a higher level of maternal education is associated with greater access to household resource and improved access to health care. Unfortunately, illiteracy rates are very high in the developing countries and as high as 37% in sub-Saharan Africa (21). Evidence shows that 39% of adults in Nigeria are uneducated (22).

Currently, a high proportion of women have barriers to accessing health care in Nigeria. For instance, NPC and ICF Macro's (23) report revealed that 56% of women experienced financial barriers to accessing health care while one-third had physical barriers. Another third had resource-related barriers. In addition, under-five mortality currently stands at 157 per 1,000 live births in Nigeria (23); hence, childhood mortality remains a major public health challenge in the country. While many studies have been conducted to understand factors driving childhood mortality in Nigeria (15, 24–27) evidence is sparse on the effects of difficult access to health care on child survival in the country. Thus, we hypothesize that barriers to accessing health care in Nigeria could be contributing to the increase in under-five mortality level in the country.

Although many studies have established the importance of access to health care in addressing poor health outcomes (28–30), how barriers to health care contribute to under-five mortality has received less attention in Nigeria. Hence, this study is designed to address this knowledge gap.

## Data and methods

Population-based cross-sectional data from the 2008 Nigeria Demographic and Health Survey (NDHS) were used in this study. The survey elicited information on demographic and health indicators at the national, regional, and state levels from a nationally representative sample of 36,800 households across the country. The primary sampling unit, which was regarded as a cluster for the 2008 NDHS, is defined on the basis of enumeration areas (EAs). The sample for the survey was selected using a stratified two-stage cluster design consisting of 888 clusters (23). Data were collected using face-to-face interviews from 33,385 women aged 15–49. Out of the survey's complete sample size of 33,385 women, the sample size for this study comprises 18,028 women who had a total of 28,647 live births within the 5 years preceding the survey. Thus, analysis was restricted to 28,647 live-born children delivered by 18,028 women within the 5 years before the survey. Birth history data, including child's sex, month and year of birth, as well as information on the child's survivorship status, were elicited for each of the live births. The study utilized the births recorde of the 2008 Nigeria DHS data.

### Variables measurement

#### Outcome variable

In this study, the outcome variable was the risk of death under the age of 5 years, defined as the probability of a live-born child dying before its fifth birthday. This is measured as the duration of survival since birth in months.

#### Explanatory variables

In terms of exposure, the main explanatory variables were: (A) ‘cultural barriers to accessing health care’; (B) ‘resource-related barriers to accessing health care’; and (C) ‘physical barriers to accessing health care’. We generated these variables using eight DHS standard questions. In the DHS women data, women were asked whether a range of factors would be a big problem for them in accessing health care. These factors included:

1) getting permission to access health care, 2) not wanting to go alone, 3) concern that a female health worker may not be available, 4) getting money needed for treatment, 5) concern that a male health worker or any health worker may not be available, 6) concern that drugs may not be available, 7) distance to health care facility, and 8) transport cost.

Responses to these questions were categorized as: 1) big problem – coded as ‘1’ and 2) not a big problem – coded as ‘0’. Firstly, we combined responses to the first three factors (stated in 1–3 above) to generate ‘cultural barrier to accessing health care’. We considered it a cultural issue for a woman to have difficulty securing their husband's permission to seek medical help. As noted earlier, women in purdah in some parts of Northern Nigeria are expected to always stay indoors, and may find it difficult to go out in extreme situations such as to get medical treatment (20). Also, concerns that female health worker may not be available, and unwillingness to go alone to seek medical help are considered as cultural barriers to access.

Secondly, responses to the fourth, fifth, and sixth factors stated above were used to generate the ‘resource-related barrier’. Thirdly, the seventh and eighth factors above were used to generate the ‘physical barrier’ covariate. An overall index (composite score) was created to typify the extent or severity of the problem in accessing health care. We achieved this by adding the dichotomous variables in all the eight situations – three situations each for (A) ‘cultural barrier’ and (B) resource barrier’, and two situations for (C) ‘physical barrier’. This process produced a minimum of ‘0’ and a maximum of ‘4’ for A and B, and a minimum of ‘0’ and a maximum of ‘3’ for C (i.e. physical barrier). A higher score on this scale describes a situation of increased difficulty in accessing health care services, while a lower score typifies less difficulty. For clarity purpose, the composite scores were later dichotomized. Respondents who had scores of 0 and 1 were categorized as having ‘less difficulty’ while those who had scores from 2 to 4 were considered as having ‘more difficulty’ in accessing health care.

Other independent variables of interest (which could influence child survival as established in the reviewed literature) are respondents’ age (categorized as 15–23, 24–34, and 35 or older); place of residence (rural and urban); educational level (grouped as no education, primary, secondary, and higher); occupation (not working, professional/technical/managerial, sales/clerical/service, and manual labor); wealth index (categorized as poorest, poorer, middle, richer, and richest); religion (Muslim, Catholic, other Christians, and others); marital status (grouped as never married, currently married, and previously married). We also considered important child's characteristics which could influence child survival as established in the literature. These include birth order (categorized as first birth, 2–4, 5+), and birth interval (categorized as less than 24 months, 24+).

### Statistical analyses

Three levels of analysis were employed in this paper. At the univariate level, the percentage distribution of the study sample was presented to show the distribution of respondents by the women and children characteristics stated above. At the bivariate level, Pearson's Chi-square test was performed to examine the statistically significant relationship between independent variables and child survival Cox proportional hazards model was employed to examine the relationship between ‘barriers to accessing health care’ and child survival, while adjusting for the effect of selected characteristics.

### Survival analysis

Cox proportional hazards model (survival analysis) is appropriate in analyzing time-to-event as an outcome variable where it can be assumed that the explanatory variables have a multiplying effect on the hazard rates. This means that, using Cox proportional hazards model, both the occurrence of under-five mortality and the time when the child died were combined to generate the outcome variable. In addition, Cox regression analysis handles the censoring problem and permits the inclusion of censored observation. In medical and social science research, an observation is said to be censored when the outcome of interest has not occurred (31).

Using Cox proportional hazards model, the probability of under-five death was regarded as the hazard. The hazard was modeled using the following:

$$H(t)=H_{0}(t)\exp.(b_{1}X_{1}+b_{2}X_{2}+b_{3}X_{3}+\cdots b_{k}X_{k})$$

where *X*_1_ … *X*_*k*_ are a collection of explanatory variables and *H*_0_(*t*) is the baseline hazard at time *t*, representing the hazard for a person with the value 0 for all the explanatory variables. By dividing both sides of equation 1 by *H*_0_(*t*) and taking logarithms, eq. 1 becomes:

$$\text{In}\left(\frac{H(t)}{H_{0}(t)}\right)=b_{1}X_{1}+b_{2}X_{2}+b_{3}X_{3}+\cdots b_{k}X_{k}$$

where *H*(*t*)/*H*_0_(*t*) is regarded as the hazard ratio. The coefficients *b*_1_ … *b*_*k*_ are estimated by Cox regression (32).

Further, to examine how ‘barriers to health care’ influence child survival, eight different models were fitted. The first model (univariate model) examines the independent effects of barriers to health care on child survival. Model 2 examines the effects of cultural barriers and children characteristics on child survival. While Model 3 examines the effects of resource-related barriers and children characteristics on the outcome, Model 4 adjusts for the effects of physical barriers and children characteristics. Further, Models 5, 6, and 7 adjust for the effects of cultural, resource, and physical barriers, respectively, on child survival, with each of the three models (5–7) incorporating women's characteristics as controls. Model 8 is the final model which considered all the selected characteristics in our analysis. All analysis was done using Stata (version 11.2). To correct for under-sampling and oversampling of some areas in the country and to ensure national representativeness, weighting factors constructed by the Measure DHS were applied in our analysis.

## Results

### Characteristics of the study sample

Table 1 presents the percentage distribution of the study population by selected characteristics. As shown in the table, results indicate that about two out of five children (37.9%) were children of mothers who reported cultural issue as a barrier to accessing health care. Almost half of the children (43.8%) belonged to mothers who reported resource-related issues as barriers to accessing health care. Also, 43.5% were children of mothers who had geographical or physical problem as barriers to accessing health care. Most of the children (46.2%) were of birth order 2–4, and the majority (80.8%) had a preceding birth interval of at least 24 months. About half of the children (46.5%) were children of mothers with no formal education, and most of the children (23.2%) were from the poorest households. More than half of the children (55.4%) belonged to Muslim mothers. A high proportion of the children (96.8%) were delivered by currently married women. About half (49.8%) were children of women aged 25–34, and 70.2% of the children were from rural area.

**Table 1:** Percentage distribution of children by their survival status and according to selected characteristics

		Child survival status (%)		
			
Variables/categories	Total (*n*=28,647); N (%)	Alive	Dead	Chi-square
Cultural issues				71.0***
Less difficulty	17,472 (62.1)	90.4	9.6	
More difficulty	10,929 (37.9)	86.4	13.6	
Resource-related issues				77.7***
Less difficulty	15,779 (56.2)	90.6	9.4	
More difficulty	12,697 (43.8)	86.7	13.3	
Geographical issues				8.8**
Less difficulty	15,599 (56.5)	89.5	10.5	
More difficulty	12,913 (43.5)	88.1	11.9	
Birth order				34.9***
First order	5,353 (19.1)	89.6	10.4	
2–4	13,069 (46.2)	90.3	9.7	
5+	10,225 (34.7)	86.5	13.5	
Birth interval				212***
Less than 24 months	5,375 (19.2)	82.5	17.5	
24 months or higher	23,272 (80.8)	90.4	9.6	
Educational level				35.7***
No formal education	14,418 (46.5)	86.8	13.2	
Primary	6,552 (23.2)	89.0	11.0	
Secondary	6,338 (24.9)	91.3	8.7	
Tertiary	1,339 (5.4)	95.4	4.6	
Mother's occupation				5.5**
Not working	9,035 (30.4)	88.8	11.2	
Prof/tech/managerial	959 (3.7)	93.5	6.4	
Sales/clerical/service	9,026 (33.7)	88.8	11.1	
Manual labor	9,471 (32.2)	88.5	11.5	
Wealth index				31.3***
Poorest	7,604 (23.2)	86.3	13.7	
Poorer	6,871 (22.8)	87.0	13.0	
Middle	5,609 (19.3)	88.4	11.6	
Richer	4,755 (17.8)	90.6	9.4	
Richest	3,808 (16.9)	93.6	6.4	
Religious affiliation				13.1***
Muslim	16,152 (55.4)	87.7	12.3	
Catholic	2,452 (9.3)	89.6	10.4	
Other Christians	9,286 (33.7)	90.6	9.4	
Others	547 (1.6)	89.5	10.5	
Mother's marital status				16.2***
Never married	506 (1.7)	91.3	8.7	
Currently married	27,378 (96.8)	89.0	11.0	
Previously married	446 (1.6)	11.0	18.3	
Mother's age				10.3***
15–24	7,249 (24.8)	88.0	12.0	
25–34	14,111 (49.8)	90.1	9.9	
35+	7,287 (25.4)	87.4	12.6	
Place of residence				64.0***
Urban	7,613 (29.8)	92.0	8.0	
Rural	21,034 (70.2)	87.6	12.4	

** *p*<0.01

*** *p*<0.001.

### Background characteristics and children's survival status

Table 1 also presents the percentage distribution of the children by their survival status and according to selected characteristics. As presented in the table, the percentage of under-five deaths was significantly highest for children whose mothers had cultural barriers to accessing health care (13.6%, *p*<0.001), followed by children whose mothers had resource-related barriers (13.3%, *p*<0.001) and those whose mothers had geographical/physical barriers to health (11.9%, *p*<0.01). In addition, under-five mortality was significantly associated with other selected characteristics in our analysis (*p*<0.001). For instance, the percentage of under-five deaths was significantly highest for children of fifth or higher birth order (13.5%), children with shorter birth interval (17.5%), children of uneducated mothers (13.2%), children from the poorest households (13.7%), children of Muslim mothers (12.3%), children of previously married women (18.3%), and children in rural areas (12.4%).

### Multivariate results

Results from the multivariate analysis are presented in Tables 2 and 3. Model 1 presents the unadjusted hazard ratios showing the independent effects of each of the ‘barriers to health care’ variable on child survival. As presented in Table 2, barriers to accessing health care were significantly associated with elevated hazards of under-five death. For instance, risk of death before age five was about two-fold higher for children whose mothers had cultural barriers to accessing health care (HR: 1.44, CI: 1.32–1.57, *p*<0.001) compared to children whose mothers reported no cultural barriers. Risk of death before reaching age five were 43% significantly higher for children whose mothers had resource-related barriers to accessing health care (HR: 1.43, CI: 1.32–1.55, *p*<0.001), compared to their counterparts whose mothers had no resource-related barriers. In addition, children of mothers who had a physical barrier to accessing health care had elevated hazards of dying before age five (HR: 1.13, CI: 1.04–1.25), relative to children whose mothers reported no physical barriers.

**Table 2:** Effects of barriers to health care and selected children characteristics on under-five mortality, Nigeria 2008 (Models 1–4)

Variables/categories	Model 1^a^	Model 2^b^	Model 3^c^	Model 4^d^
Cultural barrier to accessing health care index	UHR (CI)	AHR (CI)	AHR (CI)	AHR (CI)
Cultural issues				
Less difficulty	1	1		
More difficulty	1.44 (1.32–1.57)***	1.39 (1.28–1.51)***		
Resource-related issues				
Less difficulty	1		1	
More difficulty	1.43 (1.32–1.55)***		1.36 (1.26–1.48)***	
Geographical issues				
Less difficulty	1			1
More difficulty	1.13 (1.04–1.24)***			1.11 (1.02–1.20)*
Birth order				
First order		1	1	1
2–4		0.76 (0.68–0.85)***	0.76 (0.68–0.85)***	0.75 (0.67–0.84)***
5+		1.06 (0.94–1.18)	1.05 (0.94–1.18)	1.07 (0.96–1.20)
Birth interval				
Less than 24 months		1	1	1
24 months or higher		0.52 (0.47–0.57)***	0.51 (0.47–0.56)***	0.51 (0.47–0.56)***

* *p*<0.05

*** *p*<0.001. UHR=unadjusted hazard ratio; AHR=adjusted hazard ratio.

a Model which examines independent effects of each of the barriers to health on child survival

b effects of cultural barrier and children characteristics on child survival

c effects of resource barrier and children characteristics on child survival

d effects of physical barrier and children characteristics on child survival.

**Table 3:** Effects of barrier to health care and selected characteristics on under-five mortality, Nigeria 2008 (Models 5–8)

Variables/categories	Model 5^e^	Model 6^f^	Model 7^g^	Model 8^h^
Cultural issues	AHR (CI)	AHR (CI)	AHR (CI)	AHR (CI)
Less difficulty	1			1
More difficulty	1.29 (1.18–1.41)***			0.89 (0.81–0.98)
Resource-related issues				
Less difficulty		1		1
More difficulty		1.29 (1.19–1.40)***		1.21 (1.09–1.34)***
Geographical issues				
Less difficulty			1	1
More difficulty			0.98 (0.90–1.08)	1.18 (1.08–1.29)***
Birth order				
First order				1
2–4				0.77 (0.68–0.87)***
5+				0.97 (0.83–1.12)
Birth interval				
Less than 24 months				1
24 months or higher				0.51 (0.47–0.56)***
Educational level				
No formal education	1	1	1	1
Primary	0.94 (0.84–1.06)	0.94 (0.83–1.05)	0.92 (0.82–1.04)	0.98 (0.85–1.08)
Secondary	0.86 (0.74–1.00)	0.86 (0.74–1.00)*	0.84 (0.72–0.97)*	0.89 (0.76–1.03)
Tertiary	0.52 (0.35–0.75)**	0.52 (0.35–0.75)**	0.50 (0.35–0.73)**	0.55 (0.38–0.79)**
Mother's occupation				
Not working	1	1	1	1
Prof/tech/managerial	1.17 (0.87–1.57)	1.16 (0.86–1.56)	1.14 (0.86–1.54)	1.17 (0.88–1.57)
Sales/clerical/service	1.09 (0.98–1.21)	1.09 (0.98–1.20)	1.08 (0.98–1.20)	1.10 (0.99–1.21)
Manual labor	1.04 (0.93–1.17)	1.04 (0.92–1.17)	1.03 (0.92–1.16)	1.05 (0.93–1.18)
Wealth index				
Poorest	1	1	1	1
Poorer	0.98 (0.87–1.10)	0.98 (0.87–1.10)	0.97 (0.87–1.09)	0.96 (0.86–1.07)
Middle	0.97 (0.85–1.10)	0.96 (0.84–1.09)	0.95 (0.83–1.09)	0.95 (0.83–1.08)
Richer	0.87 (0.75–1.01)	0.87 (0.75–1.01)	0.85 (0.73–0.98)*	0.85 (0.73–0.99)*
Richest	0.71 (0.57–0.89)**	0.72 (0.57–0.89)**	0.69 (0.55–0.86)**	0.70 (0.56–0.88)**
Religious affiliation				
Muslim	1	1	1	1
Catholic	1.07 (0.92–1.25)	1.06 (0.91–1.24)	1.03 (0.88–1.21)	1.010 (0.94–1.28)
Other Christians	0.97 (0.86–1.11)	0.96 (0.84–1.09)	0.94 (0.83–1.07)	1.00 (0.88–1.14)
Others	0.81 (0.61–1.08)	0.80 (0.60–1.07)	0.81 (0.61–1.08)	0.83 (0.63–1.10)
Mother's marital status				
Never married	1	1	1	1
Currently married	1.21 (0.86–1.71)	1.21 (0.86–1.72)	1.21 (0.85–1.72)	1.22 (0.86–1.72)
Previously married	2.18 (1.43–3.29)***	2.14 (1.41–3.25)***	2.14 (1.41–3.25)***	2.22 (1.47–3.34)***
Mother's age				
15–24	1	1	1	1
25–34	0.88 (0.80–0.97)*	0.88 (0.80–0.97)*	0.88 (0.79–0.97)**	0.85 (0.76–0.95)**
35+	1.08 (0.97–1.21)	1.08 (0.97–1.20)	1.07 (0.96–1.20)	0.99 (0.86–1.14)
Place of residence				
Urban	1	1	1	1
Rural	1.22 (1.07–1.38)**	1.21 (1.07–1.37)**	1.23 (1.08–1.39)**	1.21 (1.08–1.37)**

* *p*<0.05

** *p*<0.01

*** *p*<0.001.

AHR=adjusted hazard ratio.

e Effects of cultural barrier and mothers characteristics on child survival

f effects of resource barrier and mothers characteristics on child survival

g effects of physical barrier and mothers characteristics on child survival

h effects of the 3 barrier variables and all selected characteristics on child survival.

After adjusting for the selected children characteristics in Models 2–4, results still established various barriers to health care as important predictors of under-five mortality in Nigeria. For instance, results in Model 2 showed that risk of death during the first 5 years of life was 36% higher for children whose mothers had cultural barriers to health care (HR: 1.36, CI: 1.26–1.48, *p*<0.001), compared to children whose mothers reported no cultural barriers.

Additionally, after adjusting for the effects of the selected women's characteristics in Models 5–6, findings indicated significant association between under-five mortality and cultural barriers (HR: 1.29, CI: 1.18–1.41, *p*<0.001) and resource-related barriers (HR: 1.29, CI: 1.19–1.40, *p*<0.001). However, the relationship between child survival and physical barriers to health became insignificant after adjusting for women characteristics in Model 7.

Model 8 (final model) examines the influence of all types of barriers to health care and other selected factors on child survival. Results, as presented in Model 8 (Table 3), indicate a significant relationship between child survival and resource-related barriers (HR: 1.21, CI: 1.09–1.34, *p*<0.001) and physical barriers (HR: 1.18, CI: 1.08–1.29, *p*<0.001). Cultural issues became less important after adjusting for the effects of all selected characteristics. The control variables in the final model such as birth order, birth interval, maternal education, wealth index, marital status, mother's age, and place of residence were significantly associated with under-five mortality. For instance, being a child of a mother who had tertiary education (HR: 0.55, CI: 0.38–0.79, *p*<0.01), being a child of a mother from the richest household (HR: 0.70, CI: 0.5–0.88), and being delivered after a preceding birth interval of 2 years or more (HR: 0.51, CI: 0.85–1.07) was associated with lower risks of under-five mortality. Conversely, being a child of a previously married women (HR: 2.22, CI: 1.47–3.34, *p*<0.001), and residence in a rural area (HR: 1.21, CI: 1.08–1.37, *p*<0.01) was associated with elevated risks of under-five mortality.

## Discussion and conclusion

Understanding the effect of difficult access to health care on child survival is very important at a time like this when there are growing inequalities in access to health care services in sub-Saharan Africa. Results of this study established that unhindered access to health care plays a very important role in child health and survival. Our findings indicate that child survival was significantly higher among the children of mothers who reported no barriers to accessing health care. Cultural and resource-related factors stood out as key barriers to health care utilization in Nigeria. Consequently, these were found to be associated with elevated risks of death among under-five children in the country.

Nigeria is a multi-ethnic country with diverse cultural practices (33). Different ethnic factors such as difficulty in getting permission to seek medical treatment are serious barriers to timely health care utilization in some parts of Nigeria. For instance, Wall (19) noted that Hausa women of Northern Nigeria are rarely seen in public due to an adoption of a very strict form of purdah (i.e. wife seclusion). As a result of this culture of spatial constraint, many Hausa women have limited autonomy and freedom of movement, and hence are subject to male dominance and social control. More recently, Hugo (20) noted that women in purdah are expected to always remain indoors, except in extreme situations such as to get medical treatment and that has to be with the husband's permission. Even in extreme situation such as during child labor, a woman in purdah cannot seek medical help until the husband grants permission or personally accompanies the woman to a health facility.

Due to the cultural differences among Nigerians, there are huge ethnic variations in health care utilization in the country. For example, Babalola and Fatusi (34) found that antenatal and postnatal care utilizations were lower among Hausa women compared to Igbo and Yoruba ethnic groups. Recent evidence provided by Ononokpono et al. (16) also indicates that antenatal care visits are lower among Hausa/Fulani/Kanuri women compared to other ethnic groups. This is a reflection of ethnic differences in access to health care services and utilization.

Meanwhile, a higher level of maternal education has been established as an important factor for the achievement of improved access to health care services and utilization (14). Sastry (35) noted that policies to increase child survival through the pathways of improved access to health care are likely to be more beneficial to children of better-educated mothers. This is essentially because education plays a vital role in shaping cultures, opinions, customs, norms, and attitudes, and it also determines exposure to a range of new ideas and values. Education is also the only phenomenon that can loosen an individual from traditional norms and cultural practices inimical to child health and survival. Since 39% of adult are uneducated in Nigeria (22), the attempt to increase child survival through the mechanism of improved access to health care services and utilization must include strategies aimed at increasing women education in the country.

Results of this study further suggest that resource-related barriers to health care utilization are a statistically important factor driving high under-five mortality in Nigeria. Factors such as difficulty in getting money needed for medical treatment, shortage of health workers and inadequate supply of drugs are some of the resource-related barriers identified for timely and quality health care interventions in the country. Our findings revealed that around one-quarter of the children were children of women who reported no resource-related barrier to health care utilization while an overwhelmingly high proportion (about three-quarters) were children of mothers who expressed resource-related concerns as barriers to accessing health care. Akin et al. (12) attributed the shortage of health personnel and inadequate supply of drugs to scarcity of health funds in many developing countries. Worse still and more recently, there are health budget cutbacks in many countries due to the recent economic crisis (13). Evidence suggests that the shortage of a health workforce has reached a critical level in sub-Saharan Africa and that at the current pattern of workforce training, it would take between 29 and 36 years for many countries to achieve WHO's target of 2.28 professionals per 1,000 population (36). In 2006, WHO estimated that there was a shortage of more than 4.3 million health workers globally, and sub-Saharan Africa is the hardest-hit (37). For instance, 36 out of 57 countries with critical shortages were in sub-Saharan Africa (37). Awofeso (38) noted that Nigeria is one the countries in the region faced with a health workforce crisis. Consequently, our findings suggest that the shortage of health workers has created resource-related barriers to health care services and utilization in Nigeria and, therefore, negatively influences child survival in the country.

Other reasons attributed to a shortage of health personnel in Nigeria and other developing countries include emigration of medical workers to the economically advanced nations (39, 40). Willis-Shattuck et al. (41) argue that motivational factors such as financial incentives, good career development, good working environment, and availability of adequate resources and infrastructure will be necessary to improve the morale of health workers in order to reverse the present trend in health workers emigration from the developing countries.

Further, our findings established a significant association between physical barriers to accessing health care and under-five mortality in Nigeria. Previous studies have established inequality in the distribution of health care facilities in Nigeria (42) and other parts of the developing world (5, 43). Considering the growing within-country inequality in childhood mortality (11, 23), efforts to reduce under-five mortality in Nigeria must include strategies that will ensure equitable distribution of health care facilities across regions of the country. An increase in the density of modern health care facilities, particularly in the rural areas and settings where transportation systems and road infrastructures are poor, will bring about high proximity to health care facilities.

Findings of this study clearly established that the substantial barriers to accessing health care in Nigeria negatively impact upon child health and survival. To address this public health challenge, there have been tremendous efforts by both government and non-governmental organizations in the country. For instance, at present, various state governments, such as Ondo, Osun, Oyo, Imo, Lagos States, etc., and governments at other levels in the country have developed such initiatives to mitigate physical barriers to accessing health care through the equitable distribution of health care facilities across the different parts of the country. Besides, efforts are being intensified to retain more health care personnel through the provisions of incentives, particularly for the health workers deployed in the rural areas. In addition, government at the national level is encouraging public–private partnerships to increase access to quality health care, particularly for women and their children (44) Access to health care may improve in Nigeria through such strategies as improved access to primary health care as well as increasing budgets to the health sector (45).

The finding that unhindered access to health care services is associated with significant child survival benefits has important policy implications. Policies that will improve child health and survival in Nigeria must include strategies aimed at increasing access to health care services through community-based interventions that target the elimination of barriers to health care services. Targeted interventions to address barriers to timely and quality health care services will significantly contribute towards accelerating the reduction of childhood mortality in Nigeria.

## Limitations of the study

Some limitations should be borne in mind while interpreting the findings of this study. First, the study utilized secondary data. As a result, important factors such as measurable distances to health facilities, and cultural issues like feeding and childcare practices could not be considered because of their non-availability in the DHS datasets. Further studies on barriers to accessing health care should adopt a mixed method approach to deepen the understanding of the implication of cultural concerns and health care utilization for child survival. Second, a cause–effect relationship could not be explored due to the cross-sectional nature of the DHS data.
